# Commentary on a recent review of lithium toxicity: what are its implications for clinical practice?

**DOI:** 10.1186/1741-7015-10-132

**Published:** 2012-11-02

**Authors:** Bruno Müller-Oerlinghausen, Michael Bauer, Paul Grof

**Affiliations:** 1 Drug Commission of the German Medical Association, Berlin, Germany; 2 Department of Psychiatry and Psychotherapy, University Hospital Carl Gustav Carus, Technische Universität Dresden, Dresden, Germany; 3 Mood Disorders Center of Ottawa and Department of Psychiatry, University of Toronto, Canada

**Keywords:** Lithium, bipolar disorder, renal effects, parathyroid, nephrotoxicity, thyroid, meta-analysis

## Abstract

A recent paper by McKnight *et al. *in *The Lancet *has provided the first formal meta-analysis of the more common adverse reactions to lithium. The authors analyzed 385 studies and focused mainly on the harmful effects of lithium on the kidney, the thyroid and parathyroid glands, body weight, skin and congenital malformations. Their contribution is important and welcome, but as a guide for practice, it needs to be complemented by other relevant observations and individual patient-focused perspectives.

The findings from that meta-analysis somewhat underestimate the renal side-effects, and distort to some degree or exclude other adverse effects. The glomerular filtration rate is reduced but not more than 0 to 5 ml/min/year of observation; this may not fully reflect the present state of knowledge. A quarter of patients in the study had abnormalities of the thyroid and/or parathyroid gland, and lithium was found to increase body weight significantly less than did olanzapine. Unfortunately, the authors did not consider the observations from spontaneous reporting systems, which may have changed the picture.

We feel that some specific limitations of the study were related to the inclusion of patients regardless of adequacy of treatment, quality of monitoring, drug combinations, age and sex, and stabilization response.

## Introduction

In all treatment guidelines for bipolar disorder (BD), lithium has been recommended as a first-line maintenance treatment. Some directives have gone further; recent evidence-based and consensus-based German guidelines [1] firmly endorse lithium salts as the only first-line maintenance treatment in BD, and some investigators qualify lithium as the only proven mood stabilizer [2]. However, the potential side-effects and risks of lithium treatment may at times make the implementation of these recommendations in daily practice challenging.

The adverse effects (AEs) and all practical aspects of lithium treatment are detailed in a comprehensive textbook published in 2006 [3]. In six chapters, the book summarizes the toxicological aspects of lithium treatment, and provides practical recommendations for the safe use of lithium salts in acute and long-term treatment. However, the recent paper by McKnight *et al*., a research group from Oxford [4]. has provided the first formal meta-analysis of the more common AEs to lithium. This contribution is new, important, and welcome. However, as a guide for practice, such statistical analysis, because of the limitations outlined below, needs to be complemented by other relevant observations and individual patient-focused perspectives. Taking this into account it seems, as we explain later, that the findings from this meta-analysis somewhat underestimate the renal side-effects and distort to some degree or exclude other AEs.

## Lithium and renal function

For the meta-analysis, nearly 6000 publications on various aspects of potential lithium toxicity were screened, and 385 studies published in English, French and German were included in their analysis. The report focuses on the harmful effects of lithium on the kidney, the thyroid and parathyroid, body weight, skin and congenital malformations. The authors searched primarily for controlled studies (22 randomized controlled trials (RCTs), prospective cohort studies, and case-control studies) but, in their absence, also considered prospective observations and case reports. Expanding the review beyond time-limited RCTs is important because lithium treatment is usually applied long term, and the initial and later side-effects differ. Such an inclusive approach is in accordance with the policy of most regulatory agencies, and is important for early identification of suspected AEs. Unfortunately, the authors did not consider data from national or international spontaneous reporting systems for AEs.

McKnight *et al. *[4] discuss in detail the effects of long-term lithium on renal function particularly. They found that the reduction in the glomerular filtration rate (GFR) was relatively small: 0 to 5 ml/min over each year of observation, while urinary concentration ability was on average reduced by 15%. As for long-term consequences, the authors refer to a Swedish registry showing that renal failure occurred in 18 of 3369 patients (0.5%), that is, double the incidence in the general Swedish population. McKnight *et al*. [4] conclude that 'there is little evidence for a clinically significant reduction in renal function in most patients, and the risk of end-stage renal failure is low.'

However, a meta-analysis is not equipped to assess this issue fully. Reduced GFR, or rather end-stage renal failure, only starts appearing in some patients after continuous treatment for more than 15 to 20 years, whereas meta-analyses will unavoidably include numerous patients treated for shorter periods. In this respect, more informative are the recent studies [5-8] on prolonged lithium treatment that have shown that the risk of renal end-stage failure might not be that rare, even in subjects properly managed on lithium for more than 15 years. Unfortunately, regular kidney function monitoring is often lacking in practice: a large French study shockingly showed that serum creatinine serum levels had not been performed in 40% of patients on lithium between 1997 and 2004 [9].

More informative observations from other investigators are needed. The International Group for The Study of Lithium-Treated Patients (IGSLI) has been concerned about these issues, and at present is stimulating and supporting studies in lithium centers from which data are available for important clinical variables, longitudinal courses, and renal examinations of regularly monitored patients. The findings should allow formulation of practical recommendations for the rational management of individual patients. When to discontinue lithium because of serious renal problems is a particularly vexing problem. This decision cannot be made solely by the treating nephrologist, but also requires expert psychiatric evaluation of the benefits and true risks that the individual patient can expect from his/her lithium medication in future years [10,11].

## Endocrine effects and weight gain

McKnight *et al *assessed the AEs of lithium on the thyroid, as reported by 77 studies (including 4 RCTs) with varying methodological approaches. The risk of clinical, subclinical and laboratory (as measured by thyroid-stimulating hormone levels) hypothyroidism was increased by five times. As McKnight *et al*. point out, it was difficult to include some papers published more than 30 years ago because of difficulty in comparing the laboratory resultss. The meta-analysis also found that serum parathormone and calcium levels increased by about 10%. Overall, a quarter of patients had abnormalities of the thyroid and/or the parathyroid gland. Of practical importance is the finding that stimulation of parathyroid function is likely to be more common than assumed to date. Regular monitoring of serum calcium levels should, therefore, be mandatory, and if found to be consistently raised, they should be properly managed [6].

In the meta-analysis, long-term lithium treatment was found to produce modest but significant weight gain, with an odds ratio of 1.89, and this was distinctly lower than the weight gain induced by olanzapine. Weight gain can be a problem with lithium treatment, but it is less pronounced than with the most frequently prescribed 'atypical' neuroleptics [11]. The latter are prescribed increasingly for patients with BD [12,13], and serious concernshave already been expressed about this therapeutic strategy and its unfortunate metabolic effects [14-17]. Furthermore, unlike atypical neuroleptics, lithium has not been found to induce diabetes mellitus.

McKnight *et al. *mention that hair loss was described mostly in case reports; in two RCTs, hair loss occurred in 3% to 8% of patients on lithium, compared with 0% to 6% in the placebo group. Surprisingly, McKnight *et.al*. could not find sufficient evidence for the existence of lithium-induced skin abnormalities; however, a long series of case reports (available online in the appendix of their paper) tends to contradict their statistical finding.

We feel, and McKnight *et al. *readily admit, that their meta-analysis has limitations, which include both the nature of the available data and the non-consideration of potential confounding variables, and therefore, is likely to lead to a biased estimation of important AEs. The claim by McKnight *et al *that they undertook 'a clinically informative systematic toxicity profile of lithium' is only partly fulfilled because the group failed to assess and discuss many of the AEs of lithium that are particularly important to patients and may influence compliance, such as gastrointestinal problems, tremor, cognitive impairments, ataxia, or speech disorders. To give one example: in the German spontaneous reporting system of adverse drug reactions, tremor accounted for nearly 9% of the 654 reports (received until 10 October 2008, referring to lithium mono as well as combined treatment), changed gait and ataxia for more than 10%, and confusional states for 9.5% (Drug Commission of the German Medical Association, personal communication).

Below, we discuss several specific issues that need to be considered critically.

1. Practicing clinicians are primarily interested in the potential AEs experienced by properly treated patients, that is, patients who are correctly selected for long-term lithium treatment, are maintained on a minimum effective dosage, and are regularly monitored and managed. In this context, lithium treatment generates AEs in a relatively small proportion of patients. However, the reports included in the meta-analysis unavoidably included numerous patients with bipolar spectrum disorders for whom lithium treatment was not the optimal treatment choice [18], and who were treated by physicians with insufficient experience in lithium treatment. Hence, an important aspect of future work would be to compare the rates of side-effects in specialized programs as opposed to general psychiatric practice.

2. Sufficient information about dosage and serum levels of lithium could not be taken into account although the authors made efforts to exclude patients with pre-existing lithium intoxication. Many side-effects, including endocrine and metabolic effects are dose-dependent, and therefore can often be controlled by proper dose titration. Details on patient compliance and the time intervals between lithium plasma levels and AEs would have been important background information, and their absence raises questions about the validity of the data and the statistical processing. This criticism was also raised by Malhi and Berk [19] in the same issue of the journal. As clinicians, we often come across referred patients whose dosage has not been adjusted for many years, and they frequently experience side-effects because their lithium levels are frequently much higher than 1.0 mmol/l.

3. The potential confounding factors of age and sex on lithium treatment could not be considered adequately in the meta-analysis.

4. Another important piece of information for the correct interpretation of side-effects is the patient's clinical response to long-term lithium treatment. Non-responders develop side-effects distinctly more frequently than responders, even at comparable serum lithium levels [20].

5. There is also the problem of potentially harmful co-medication. McKnight *et al. *did not elaborate on whether co-prescription of potentially nephrotoxic drugs (for example, antibiotics, cyclooxygenase-2 inhibitors) or compounds that can change the pharmacokinetics of lithium was controlled for, as this can be a strong potential confounding factor particularly with regard to renal toxicity.

6. Finally, it remains unclear whether AEs such as impaired thyroid function occurred during lithium medication given alone or were seen during treatment with thyroxin supplementation.

## Conclusions

McKnight *et al. *have published an important and ambitious study, which provides statistical data that were previously not available. The paper contributes significantly to the understanding of some potential AEs of long-term lithium treatment. However, for clinical practice, the findings of this meta-analysis must be integrated with other relevant observations including those from specialized lithium centers and from national and international spontaneous reporting systems, and caution is needed because of the understandable limitations of the data available for meta-analyses.

## Competing interests

The authors declare that they have no competing interests.

## Authors' contributions

MB initiated development of this commentary, organized the correspondence between authors, journal and IGSLi members and takes overall responsibility for the content of this paper. BMO and PG drafted and wrote the manuscript, and edited the comments from the IGSLi members. All authors have read and approved the manuscript for publication.

## Authors' information

All authors and members of the IGSLi group [21] have long experience of lithium research. Furthermore, they have been involved managing specialized lithium clinics affiliated to their academic institutions. BMO and PG were founders of the IGSLi group in 1988, together with the pioneer of lithium treatment, Professor Mogens Schou. MB is the current president of IGSLi, a post held since 2002. BMO has been the chairman of the German Drug Commission for 12 years, and has a special interest and expertise in all aspects of drug safety.

## Pre-publication history

The pre-publication history for this paper can be accessed here:

http://www.biomedcentral.com/1741-7015/10/132/prepub
